# Effect of Amperage and Field of View on Detection of Vertical Root Fracture in Teeth with Intracanal Posts

**DOI:** 10.7508/iej.2016.03.011

**Published:** 2016-05-01

**Authors:** Yaser Safi, Sepanta Hosseinpour, Alireza Aziz, Masoud Bamedi, Mahsa Malekashtari, Zahra Vasegh

**Affiliations:** a *Oral and Maxillofacial Radiology Department, Dental School, Shahid Beheshti University of Medical Sciences, Tehran, Iran; *; b * Students' Research Committee, Dental School, Shahid Beheshti University of Medical Sciences, Tehran, Iran;*; c * Oral and Dental Disease Research Center, Zahedan University of Medical Sciences, Zahedan, Iran; *; d * Dentist, Tehran, Iran*

**Keywords:** Amperage, Cone-Beam Computed Tomography, Field of View, Metal Artifact, Vertical Root Fracture

## Abstract

**Introduction:**

This study sought to assess the effects of amperage (mA) and field of view (FOV) on intracanal metal post artifacts and the diagnostic parameters for detection of vertical root fracture (VRF) in teeth with intracanal posts.

**Methods and Materials:**

In this diagnostic study, 80 human single-canal teeth were evaluated by cone-beam computed tomography (CBCT). Nickel chrome cast posts were placed into root canals after root canal therapy and canal preparation. In the test group, fracture was induced by an instron machine while no fracture was induced in the control group. Deterministic and probabilistic sensitivity and specificity values at different exposure settings were statistically analyzed using the one-way ANOVA and pairwise comparisons were performed by Tukey’s test.

**Results:**

Significant differences were found between the two groups in terms of deterministic (*P*<0.0001) and probabilistic (*P*<0.013) sensitivity and deterministic (*P*<0.037) and probabilistic (*P*<0.0001) specificity at different FOV and mA combinations.

**Conclusion:**

A smaller FOV and lower mA should be preferably used for detection of VRFs in teeth with intracanal posts.

## Introduction

Vertical root fracture (VRF) refers to longitudinal fracture of the root in endodontically treated teeth originating from the apex and extending coronally [1, 2]. These fractures are among the most common causes of endodontic treatment failure; however, their detection is still challenging [3, 4]. The incidence of VRF varies from 3.7% to 30.8% [1, 5-7]. Maxillary and mandibular premolars and the mesial root of the mandibular molars are the most susceptible roots to VRF [8, 9]. Bitewing radiography, illustration, direct observation and clinical examination using a surgical microscope are the currently common techniques for detection of VRFs. However, all these methods have limited success [5, 9]. Radiographic detection of VRF is feasible based on the two following signs: Observation of a thin, radiolucent fracture line in dentin if oriented parallel to the path of beams [10] and presence of bone loss around the tooth crown or root [9].

The efficacy of medical computed tomography (CT) and cone-beam computed tomography (CBCT) has been evaluated for detection of VRFs and it has been demonstrated that three-dimensional (3D) techniques have a higher diagnostic value than convectional radiography for detection of VRF [11-14]. CBCT systems have been designed for imaging of the craniofacial region and have the advantage of lower dose compared to medical CT, limited field of view (FOV), higher accuracy and greater resolution of images and quick scanning time [15]. Moreover, CBCT provides excellent tissue contrast and eliminates burning and overlap by the adjacent teeth. Lower cost and significantly decreased patient radiation dose compared to medical CT are among other advantages of CBCT [14, 16]. Also, it has been reported that CBCT has higher accuracy for detection of VRFs compared to other imaging systems [16, 17].

Imaging of endodontically treated teeth with intracanal metal posts has always been problematic mainly due to metal artifacts [15]. Image artifact is the main factor compromising the quality of CBCT scans. Artifact is defined as any distortion or error in image not related to the object. Based on their etiology, artifacts can be categorized into several groups. Artifacts may also be caused by the limitations related to the physical process of obtaining CBCT data [18, 19].

In the clinical setting, it is recommended to decrease the FOV, change patient’s head position or separate dental arches to avoid scanning of the areas susceptible to beam hardening (*i.e.* metal restorations, metal posts and dental implants). Beam hardening as one of the most causes of artifacts, is commonly related to metals even light metals like titanium, at the conventional kilovoltages applied in CBCT [20-22]. In fact, these artifacts are created as the result of the difference in densities of the metal objects and the surrounding tissues. In some cases, artifacts can result in loss of data and compromise accurate diagnosis [18].

In order to achieve the best exposure setting for accurate detection of VRF, the effects of parameters like amperage (mA) and field of view (FOV) on the diagnostic accuracy of CBCT must be evaluated. Thus, this study sought to assess the effect of mA and FOV on diagnostic parameters of detecting VRFs in CBCT images of teeth with intracanal metal posts.

## Materials and Methods

The study protocol was approved by the Ethics Committee of Shahid Beheshti University, School of Dentistry. (Grant No.: 160). In this study, 80 extracted sound, single-canal human premolars were selected using convenience sampling. The teeth were selected by direct observation irrespective of the age or sex of patients or reason for extraction. Extracted teeth were cleaned and the anatomical crowns were cut at the level of cementoenamel junction to eliminate errors due to enamel fracture [23]. All teeth received root canal therapy and the coronal third of the root canals were preflared using #2 or 3 Gates Glidden drills (Dentsply Maillefer, Ballaigues, Switzerland). Canal preparation was continued using #15-50 hand K-files (Dentsply Maillefer, Ballaigues, Switzerland). Intracanal debris was removed by irrigation. Root canals were filled with gutta-percha (AriaDent, Tehran, Iran) and AH 26 root canal sealer (Dentsply, De Trey, Konstanz, Germany). 

One week later, post space was prepared by removing the gutta-percha from the coronal two third of the root canals using #2 or 3 Peeso reamers (Dentsply Maillefer, Ballaigues, Switzerland). In order to fabricate cast posts, a post pattern was prepared in the coronal two thirds of the canal by Duralay acrylic resin (AriaDent, Tehran, Iran) for all teeth. Nickel chrome cast posts were fabricated from this pattern. Each post was tried in the root canal and modified to achieve perfect fit. Posts were then placed into the root canal; but due to the risk of cement flow into the fracture line, cementation was not performed [23]. Each tooth was covered with a layer of wax with approximately 1 mm thickness and separately mounted in self-polymerizing acrylic blocks (Acropars, Marlic Co., Tehran, Iran) to ensure no gap formation in the root, facilitate the removal of teeth from their respective blocks and prevent separation of broken pieces following the induction of fracture [24]. Eighty teeth were randomly divided into two groups (*n*=40). In the test group, root fracture was induced by instron testing machine (Z010, Zwick GmbH, Ulm, Germany). No fracture was induced in the control group. The instron machine applied gradually increasing compressive load until the fracture sound was heard. Upon fracture, the load was immediately discontinued according to the diagram displayed on the system monitor. Cases in which load application caused root splitting, were excluded from the study and replaced with new specimens according to the inclusion and exclusion criteria.

For gold standard determination of VRFs after imaging, 1% methylene blue solution was used and the results were recorded. For this purpose, the teeth were removed from the acrylic blocks and immersed in 1% methylene blue solution. In case of fracture, dye would penetrate into the fracture line and visualize it in the form of a dark blue line on the root surface. Presence or absence of VRF was determined in all specimens as such. 

All specimens were stored in a hydrated environment during the entire course of study and were only removed from this environment at the time of intracanal post fabrication, induction of VRF and imaging.

The teeth were then randomly placed in a wax model of mandible filled with water in groups of eight. This aqueous environment was used to simulate intraoral conditions. The teeth were radiographed by Scanora 3D CBCT (Soredex, Helsinki, Finland) with 10×7.5 mm FOV at 13 mA, 13×14.5 mm FOV at 4 mA and 13×14.5 mm FOV at 13 mA. Other exposure settings remained constant (0.25 mm voxel size and 90 kVp).

Two blinded oral and maxillofacial radiologists evaluated the CBCT scans in axial, coronal and sagittal planes for the presence or absence of VRFs. The observers were allowed to adjust the contrast and brightness of images and no time limitation was set for observation of images. All scans were observed in LG Flatron W17652s monitor with 1440×90 pixels resolution.

The observers recorded their opinion regarding the possibility of VRFs based on their observations using the following scale: *definite* *presence* of VRF, *probable* *presence* of VRF, *definite absence* of VRF, *probable absence* of VRF and *non-diagnostic.*

Data were analyzed using the SPSS software (SPSS version 18.0, SPSS, Chicago, IL, USA). The intra-rater assessment data were analyzed with the weighted kappa statistics in order to assess the level of agreement. Diagnostic parameters including deterministic sensitivity (definite presence of VRF) and probabilistic sensitivity (definite and probable presence of VRF) and deterministic specificity (definite absence of VRF) and probabilistic specificity (definite and probable absence of VRF) of the observations were calculated and the results at different exposure settings (in terms of FOV and mA) were statistically analyzed using the one-way ANOVA. The Tukey’s test was applied for pairwise comparison of FOV and mA combinations for each diagnostic parameter. Type one error was considered as 0.05. 

## Results

Tables 1 and 2 show the diagnostic parameters for presence or absence of VRFs in specimens. The kappa value for intra-rater agreement was greater than 0.8 for all examiners. Comparison of the three different combinations of FOV and mA in terms of sensitivity and specificity was conducted with one-way ANOVA; which revealed significant differences in deterministic sensitivity and probabilistic specificity among groups (*P*<0.05).

***Deterministic sensitivity***

One-way ANOVA revealed a significant difference in this parameter among the three tested protocols (*P*<0.001). Multiple comparison by Tukey’s HSD test revealed that deterministic sensitivity followed this pattern in descending order: 7.5×10 mm FOV/13 mA>13×14.5 mm FOV/4 mA> 13×14.5 mm FOV/13 mA. The differences were significant for both comparisons (*P*<0.001). According to Tukey’s HSD test, this parameter in 7.5×10 mm FOV was significantly higher than 13×14.5 mm FOV and 13 mA (*P*<0.001). According to this test, this parameter in 4 mA was significantly higher than that in 13 mA (*P*<0.001).

***Probabilistic sensitivity***

One-way ANOVA revealed a significant difference in this parameter among the three tested protocols (*P*=0.013) and multiple comparisons by Tukey’s HSD test demonstrated that this parameter in 7.5×10 mm FOV and 13 mA was significantly higher than 13×14.5 mm FOV and 4 mA (*P*=0.011). However, this parameter was not significantly different between 13×14.5 mm FOV and 13 mA and the other two protocols (*P*>0.05).

It may be stated that no significant change occurred in this parameter by using different FOVs. This parameter showed no significant change by altering the amperage. 

***Deterministic specificity***

One-way ANOVA revealed a significant difference in this parameter among the three tested parameter combinations (*P*=0.037). Multiple comparison by Tukey’s HSD test revealed that this parameter was significantly higher in 7.5×10 mm FOV and 13 mA was higher than that in 13×14.5 mm FOV and 13 mA (*P*=0.047); but for 13×14.5 mm FOV and 4 mA this parameter was not significantly different from the other two protocols (*P*>0.05). According to Tukey’s test, this parameter in FOV=7.5×10mm was significantly higher than that in FOV=13×14.5mm (*P*=0.047). No significant change occurred in this parameter by changing the mA (*P*>0.05).

***Probabilistic specificity***

**Figure 1:**
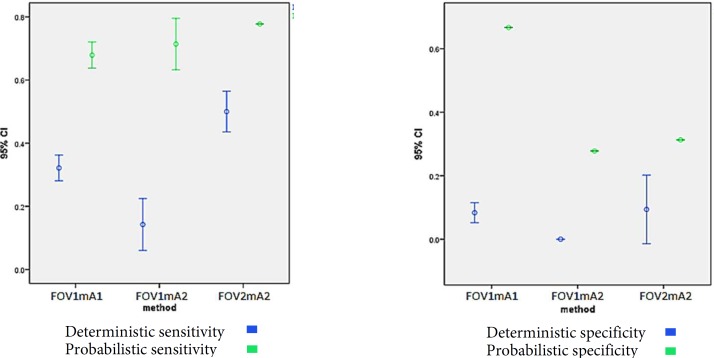
*A) *Range of deterministic and probabilistic sensitivity at different FOV and amperage (mA) combinations, *B)* Range of deterministic and probabilistic specificity at different FOV and mA combinations (FOV1=13×14.5, mA1=4, FOV1=7.5×10, mA1=13

One-way ANOVA revealed a significant difference in this parameter among the three tested protocols (*P*<0.001). Multiple comparison by Tukey’s HSD test revealed that this parameter in 13×14.5 mm FOV and 4 mA was significantly higher than 7.5×10 mm FOV and 13 mA (*P*<0.001); and the latter was significantly higher than 13×14.5 mm FOV and 13 mA (*P*<0.001). According to this test, this parameter in 7.5×10 mm FOV was significantly higher than that in 13×14.5 mm FOV (*P*<0.001) (Figure 1).

## Discussion

Detection of VRFs in endodontically treated teeth is a challenge for the clinicians because the clinical and radiographic signs and symptoms of VRF are not pathognomonic and mimic endodontic failure and periodontal lesions [24, 25]. VRFs often remain undetected during root canal therapy and eventually result in resorption, pain and dysfunction of the affected tooth [26]. Delayed or no diagnosis may necessitate invasive surgeries or result in tooth extraction. Thus, early diagnosis is critical for both the patient and clinician [27, 28].

Despite the superiority of CBCT over other imaging modalities [18, 29-33], the presence of filling materials and posts in the root canal can potentially affect the detection of root fracture; however, this effect has not been extensively studied. In 61.7% of cases diagnosed with VRF, a post is present in the root canal [25]. On the other hand, the effects of CBCT exposure parameters, particularly mA on detection of VRF in presence of intracanal posts have yet to be fully investigated. Thus, this study was undertaken to assess the effects of mA and FOV on the diagnosis of VRF in presence of a metallic intracanal post.

Calculation of accuracy and its comparison with the gold standard under *in vitro* conditions, do not provide a correct estimate of the actual diagnostic accuracy. Moreover, considering the low power, not demonstrating independent changes and occasionally contradictory values for sensitivity and specificity, *in vitro *settings may cause errors in results. Thus, in the current study, accuracy was not calculated. 

**Table 1:** Diagnostic parameters based on different FOV (mm) and amperage (mA) combinations

**Observer **	**FOV **	**mA**	**Deterministic** **Sensitivity**	**Probabilistic ** **Sensitivity**	**Deterministic ** **Specificity**	**Probabilistic** **Specificity**
**First**	13×14.5	4	0.286	0.715	0.056	0.667
**Second**	13×14.5	4	0.357	0.643	0.111	0.667
**First**	13×14.5	13	0.214	0.643	0.0	0.278
**Second**	13×14.5	13	0.071	0.785	0.0	0.278
**First**	7.5×10	13	0.444	0.777	0.0	0.313
**Second**	7.5×10	13	0.556	0.778	0.188	0.313

According to a study by Hassan *et al.* [32] the most suitable plane to confirm VRF is the axial plane. In the current study, observers first used the axial plane sections and then referred to other planes for detection of VRF. Also, observers were allowed to use enhancement filters and adjust the magnification, contrast, and *etc.* at any time. 

Kutsumata *et al.* [34], reported that voxel size, exposure settings, FOV, and particularly type of imaging system and detector, all affected the detection of VRF. On the other hand, according to Costa *et al.* [35], the magnitude of the effect of these parameters is variable between different CBCT units or in a single unit between different imaging protocols. Metska *et al. *[27], demonstrated that the accurate detection of VRFs in presence of intracanal posts depends on the type of imaging system. Since only one CBCT system was used in the current study, the effect of this factor on the results was controlled. Hassan *et al.* [36], evaluated five different CBCT systems and demonstrated that metal artifacts, noise and contrast were lower and the resolution was higher in systems using flat panel detectors compared to those using image intensifier tubes/CCD detectors. Similarly, flat panel detectors were used in this study. 

Melo *et al. *[23], and da Silveria *et al. *[24], independently evaluated the proper voxel size for detection of VRFs considering low patient radiation dose and adequate diagnostic accuracy and reported 0.2 mm voxel size as the most suitable protocol. Thus, in the current study 0.25 mm voxel size was used. On the other hand, based on these studies and our CBCT system limitations different FOVs and mAs were chosen to recognize the VRFs.

Estrela *et al.* [37], assessed the effect of type of intracanal post on the amount of artifact generated and indicated that gold and silver alloys caused the highest and carbon fiber posts created the lowest amount of artifacts. In our study, nickel chrome intracanal posts were used and produced significant amount of artifacts, which have definitely played a role in reduction of sensitivity and specificity values.

Costa *et al.* [35, 38] evaluated the effect of FOV on detection of VRF and reported that larger FOV decreased the diagnostic accuracy for detection of VRFs in presence and absence of posts. Moreover, a very low agreement existed among observers. Small FOV in absence of intracanal posts enhanced accurate detection of VRFs; but presence of intracanal posts decreased this accuracy [35, 38]. In the current study, decreasing the FOV increased sensitivity and specificity values; thus, it may be concluded that decreasing the FOV increases the accuracy of imaging. 

In an *in vitro* study, Kamburoglu and Kursun [39] demonstrated that high and very high resolutions of two CBCT systems provided higher diagnostic accuracy compared to low resolution for detection of internal resorption lacuna measuring 0.5 mm in diameter. Moreover, Haiter-Neto *et al.* [40], evaluated the diagnostic accuracy of two CBCT systems for detection of dental caries at different FOVs (6, 9 and 12 inches) and discussed that type of CBCT and FOV significantly affected the sensitivity and specificity values for detection of tooth caries.

Hedesiu *et al.* [41], found no significant difference in terms of diagnostic accuracy for detection of apical lesions at different FOVs. However, it must be noted that their study was an animal model on periapical lesions. 

Attention must be paid to the combination of FOV and mA in order to minimize the patient radiation dose. Undoubtedly, if the desired diagnostic goals can be achieved using a smaller FOV and lower mA, changing these parameters and consequently, increasing the patient radiation dose will not be acceptable at all. On the other hand, due to the possibility of false positive results, CBCT must be employed as an adjunct imaging in clinical observations in order to achieve accurate diagnosis.

Martin Palomo *et al.* [42], investigated the effects of CBCT exposure settings on patient radiation dose and reported that decreasing the voltage from 120 to 100 kVp decreased the total radiation dose by 0.62 times. Moreover, they demonstrated that decreasing the FOV diminished the radiation dose by 5-10%. The current study used 90 kVp voltage. Also, by decreasing the FOV, the diagnostic accuracy for detection of VRFs improved; in this condition, the radiation dose would decrease as well.

CBCT has greatly enhanced the clinical diagnosis in endodontics. However, it should be noted that the effective

**Table 2:** The mean deterministic and probabilistic sensitivity and specificity values in different FOV and amperage combinations of CBCT

**Diagnostic parameter (N)**	**FOV (mm)**	**Amperage (mA)**	**Mean (SD) **
**Deterministic Sensitivity (2)**	13×14.5	4	0.3115 (0.038)
	13×14.5	13	0.1425 (0.078)
	7.5×10	13	0.5 (0.061)
**Probabilistic Sensitivity (2)**	13×14.5	4	0.6790 (0.039)
	13×14.5	13	0.7140 (0.077)
	7.5×10	13	0.7775 (0.000)
**Deterministic Specificity (2)**	13×14.5	4	0.0835 (0.030)
	13×14.5	13	0 (0.0)
	7.5×10	13	0.094 (0.102)
**Probabilistic Specificity (2)**	13×14.5	4	0.667 (0.0)
	13×14.5	13	0.278 (0.0)
	7.5×10	13	0.313 (0.0)

radiation dose of CBCT is higher than that of conventional intraoral and panoramic radiographies. Thus, conventional radiography must be the first choice of clinicians for detection of VRFs due to its easy accessibility, lower cost and patient radiation dose. When a VRF is suspected, periapical radiographies must be obtained. The horizontal angulation may be changed to increase the likelihood of observing the fracture line. Eventually, if a final diagnosis cannot be made based on this conventional modality, CBCT may be indicated [24]. 

*In vitro* studies, have some limitations related to the method of creating fracture and the medium in which the teeth are stored. Thus, their results cannot be directly generalized to the clinical settings. Future studies are required to assess the diagnostic parameters at different FOV and amperage combinations for detection of VRFs in teeth without intracanal posts.

## Conclusion

In general, decreasing the FOV improves the detection of VRFs; if accompanied by a reduction in amperage, this combination would yield a more accurate diagnosis in comparison with other protocols. As stated earlier, decreasing the amperage affects the definite detection of VRFs; however, decreasing the FOV is more efficient to ensure absence of VRFs. Decreasing the amperage has a superior efficacy for overall detection of sound teeth.
